# Control of the asymmetric growth of nanowire arrays with gradient profiles

**DOI:** 10.1039/d1ra04198c

**Published:** 2021-07-28

**Authors:** Juan Patiño Cárdenas, Armando Encinas, Rossana Ramírez Villegas, Joaquín de la Torre Medina

**Affiliations:** Instituto de Investigaciones en Materiales – Unidad Morelia, Universidad Nacional Autónoma de México Antigua Carretera a Pátzcuaro No. 8701 Col. Ex Hacienda de San José de la Huerta C. P. 58190 Morelia Mexico delatorre@materiales.unam.mx; División de Materiales Avanzados, Instituto Potosino de Investigación Científica y Tecnológica A. C. Caminio a la Presa 2055 78216 San Luis Potosí, SLP Mexico

## Abstract

A novel electrochemical methodology for the growth of arrays of Ni and Co nanowires (NWs) with linear and non-linear varying micro-height gradient profiles (μHGPs), has been developed. The growth mechanism of these microstructures consists of a three-dimensional growth originating from the allowed electrical contact between the electrolyte and the edges of the cathode at the bottom side of porous alumina membranes. It has been shown that the morphology of these microstructures strongly depends on electrodeposition parameters like the cation material and concentration and the reduction potential. At constant reduction potentials, linear Ni μHGPs with trapezoid-like geometry are obtained, whereas deviations from this simple morphology are observed for Co μHGPs. In this regime, the μHGPs average inclination angle decreases for more negative reduction potential values, leading as a result to more laterally extended microstructures. Besides, more complex morphologies have been obtained by varying the reduction potential using a simple power function of time. Using this strategy allows us to accelerate or decelerate the reduction potential in order to change the μHGPs morphology, so to obtain convex- or concave-like profiles. This methodology is a novel and reliable strategy to synthesize μHGPs into porous alumina membranes with controlled and well-defined morphologies. Furthermore, the synthesized low dimensional asymmetrically loaded nanowired substrates with μHGPs are interesting for their application in micro-antennas for localized electromagnetic radiation, magnetic stray field gradients in microfluidic systems, non-reciprocal microwave absorption, and super-capacitive devices for which a very large surface area and controlled morphology are key requirements.

## Abstract 2

A novel electrochemical methodology for the growth of arrays of Ni and Co nanowires (NWs) with linear and non-linear varying micro-height gradient profiles (μHGPs), has been developed.

## Graphical abstract

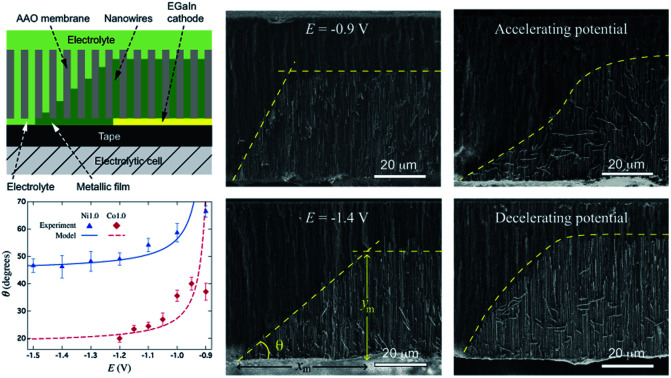

## 1 Introduction

The shift from two to three dimensions (3D) in nano-architectures is a promising avenue to develop a new generation of physicochemical and biological multifunctional device applications. The structuring of 3D systems at the nanoscale has extensive advantages because size-dependent physical effects and phenomena that are not observed at the macroscale can be exploited and combined with biochemical capabilities for the design of functional applications.^1–4^ Furthermore, 3D nanostructuration using inexpensive bottom-up synthesis approaches for mass production still represents a great challenge. In this sense, the quest for novel synthesis methods of macroscopical systems made of nanoscale building blocks with 3D resolution has attracted considerable attention and motivated intense research activity. Particularly, a special kind of 3D nano-architectures and devices that are currently being developed by many research groups are gradients of some effect or material.^5^ That is, the continuous variation of a physical/chemical property as a function of time or along a specific spatial direction leads to the generation of a gradient of the same or other property or effect. Different methods for the fabrication of gradients have been developed depending on the technological application being aimed at, as for instance compositionally gradient electrodes fabricated by selective potential-pulse electrodeposition,^6^ polymer brush gradients synthesized by catalyst diffusion,^7^ gradient polymer nanocomposites by magnetophoresis and capillary electrophoresis,^8,9^ patterned inverse opals by selective photolysis modification process,^10^ gradient plasmonic nanostructures by physical vapor deposition on curved nanomasks,^11^ wettability gradients made by electrochemical polymerization of pyrrole arrays,^12^ silicon geometric gradients by colloidal lithography,^13^ functionality gradients with thickness graded profiles by dip-coating process^14^ and gradient nanoclusters prepared by wireless electro-functionalization.^15^ Gradients for the study of biological systems are receiving special interest, for instance, cell-culturing materials^16^ and regulation of cytosolic pH.^17^

Besides these approaches, electrochemical methods have been widely used because of its low cost, reliability and ease of adaptation for the production of a large variety of materials. Particularly, bipolar electrochemistry is a promising technique for the generation of metal composition gradients with electrocatalytic activity and optical and electronic properties,^18–20^ gradient polymer surfaces for electrochemical patterning applications^21^ and surface-wetting gradients with controlled hydrophilic behavior.^22^ Although a variety of methods such as magnetron plasma aggregation, spin coating, and centrifugation have been developed to produce nanoparticle size gradients,^23–25^ electrochemical methods have been proved to be very reliable for synthesizing nanoparticle size gradients^26–28^ and concentration gradient nanowire (NW) arrays.^29^ Arrays of NWs with height gradient profiles^30^ and nanotube size gradients^31^ are microstructures based on nanoscale building blocks of elongated shapes and varying sizes synthesized by electrochemical methods, which are interesting as microwave devices^32^ and energy storage^33^ applications. Indeed, the elongated cylindrical geometry of these nanostructures is susceptible to the appearance of confinement and size-dependent effects and quantum mechanical optical, magnetic, and electrical properties.^34–37^ Other systems consisting of arrays of programmable stimuli-responsive hybrid magnetic micropillars, polydimethylsiloxane micropillars, and slanted functional gradient micropillars have been proposed as novel prototypes for reconfigurable patterns upon actuation,^38,39^ microfluidics^40^ and bioinspired dry adhesives as self-cleaning superhydrophobic and biosensing applications,^41^ respectively. Previous works on the use of a combined technique based on electrodeposition and dip-coating have been developed to synthesize gradients with transversal section widths of the order of 10^2^–10^3^ μm.^14,30^ However, these techniques do not allow the synthesis of gradients of lateral widths of the order of several micrometers wide.

In this work, we have developed a novel room temperature electrochemical methodology for the growth of fixed diameter NW-based 3D microstructures with controlled μHGP morphology. This methodology takes advantage of the different growth dynamics that take place outside the pores and within them *via* an enhanced or reduced ionic mobility. The strategy used for the growth of these microstructures consists in allowing the lateral growth of Eutectic–Galium–Indium (EGaIn) cathode that serves for the NWs growth inside the pores of anodic aluminum oxide (AAO) or alumina (Al_2_O_3_) membranes. Such a horizontal or lateral growth generates new nucleation sites for the growth of other NWs at different initial moments. We have shown that both the material and concentration of metallic cations in solution significantly influence the morphology of μHGPs grown at constant reduction potential. Furthermore, by varying the reduction potential over time in a controlled way, it also leads to changes in the morphology of μHGPs. The proposed methodology in this work allows the synthesis of novel spatially modulated 3D microstructures based on NW building blocks, which are interesting for potential applications as site-selective application of electric potentials,^21^ non-reciprocal microwave absorption,^30^ electron field emission,^42^ and super-capacitive device applications.^43^ Furthermore, the morphology of the proposed μHGPs may also be interesting for energy conversion,^44^ optoelectrical,^45^ and thermoelectric energy conversion^46^ applications that take advantage of NWs length-dependent properties.

## 2 Experimental

Arrays of Ni and Co NWs have been grown by a standard three-probe electrodeposition technique into the pores of commercial 60 μm thick AAO membranes (Whatman, Inc.). The nominal pore diameter and porosity are 200 nm and 40%, respectively. Electrodeposition has been carried out in chronoamperometry mode by using a source measure unit (SMU) 2450 from Keithley with applied constant or variable reduction potentials in the range from −0.9 V to −1.5 V *versus* Ag/AgCl.

**Fig. 1:**
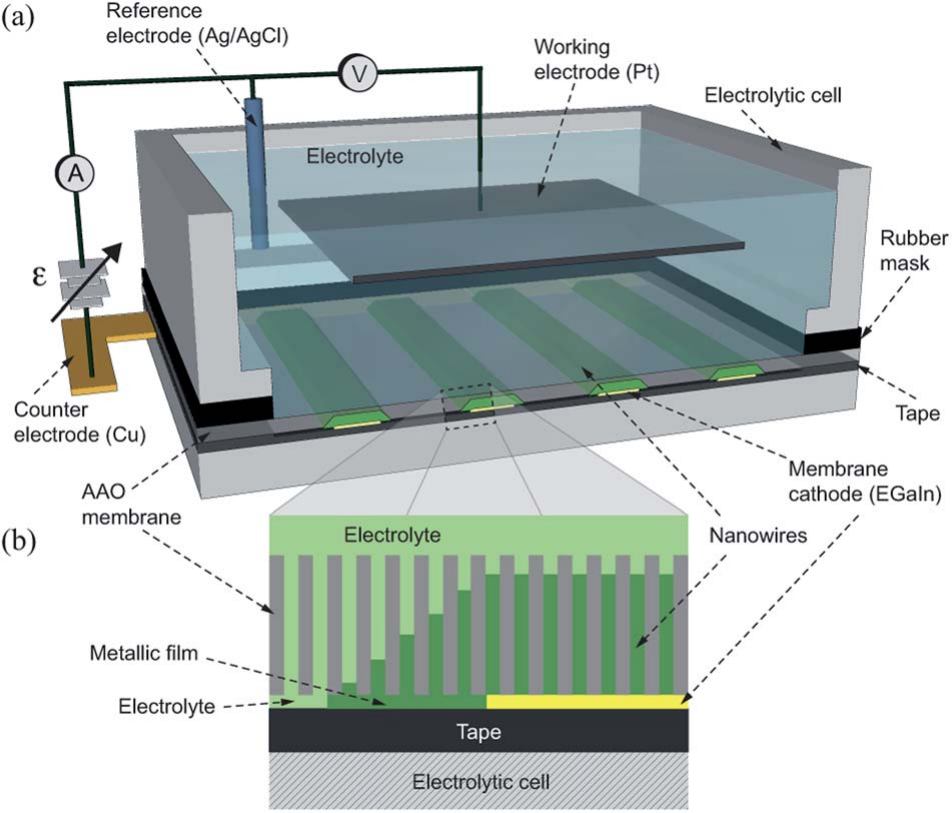
(a) Schematics of the 3D and cross section views of the three probe electrodeposition setup for the growth of μHGP. Four straight EGaIn lines are painted on the lower surface of the porous AAO membrane which is placed inside the electrolytic cell making electrical contact with the counter electrode. The cell is filled with an electrolyte to allow physical contact with the EGaIn lines through the pores of the AAO membrane. (b) Schematics of a close cross section view of the AAO membrane displaying the metallic film growing in the horizontal direction from the EGaIn cathode edges which serves as new nucleation sites for the growth of the corresponding μHGP.

Electrolytes with compositions *x*NiSO_4_·6H_2_O + 0.5 M H_3_BO_3_; and *x*CoSO_4_·7H_2_O + 0.5 M H_3_BO_3_, with the metallic cation concentrations (*x*) adjusted to 0.5 M or 1.0 M and the pH adjusted to 4, were used for the growth of Ni and Co NWs. The corresponding electrolytes for Ni (Co) NWs are named hereafter as the Ni-0.5 M (Co-0.5 M) and the Ni-1.0 M (Co-1.0 M) solutions. Prior to electrodeposition four 300–400 μm wide and 1 cm long thin straight layers of EGaIn from Aldrich are painted onto one side of the membrane to serve as cathodes for the growth of arrays of NWs with μHGP. The four EGaIn lines are painted using a cotton swab on the AAO membranes through a paper mask, thus generating eight μHGPs which serve to carry out statistics of their morphology. Then the membrane is placed on a flat surface, consisting mainly of adhesive tape that insulates the membrane along with the electrolyte and a large section of the EGaIn lines. One end of the EGaIn lines at tiny sections are in electrical contact with a flat copper conductor which is isolated from the electrolyte and serves as the counter electrode of the electrolytic cell, as shown in Fig. 1 (a). The remaining section of the EGaIn cathode lines is exposed to the electrolyte along with the other side of the porous membrane, as shown in the zoomed scheme in Fig. 1(b). As seen, exposition of the EGaIn cathode lines beneath the AAO membrane promotes a horizontal or lateral growth from their edges, leading to thin metallic layers which progressively generate new nucleation sites for other NWs. A large surface area Pt working electrode is used to ensure as far as possible a homogeneous electric field with the EGaIn lines. After electrodeposition, the EGaIn lines are removed using isopropanol to carry out the structural characterization of the samples. The NWs height and the μHGP morphology were confirmed by scanning electron microscopy (SEM) observations from the cross section of the different samples.

## 3 Results and discussion

### 3.1 Growth mechanism

**Fig. 2:**
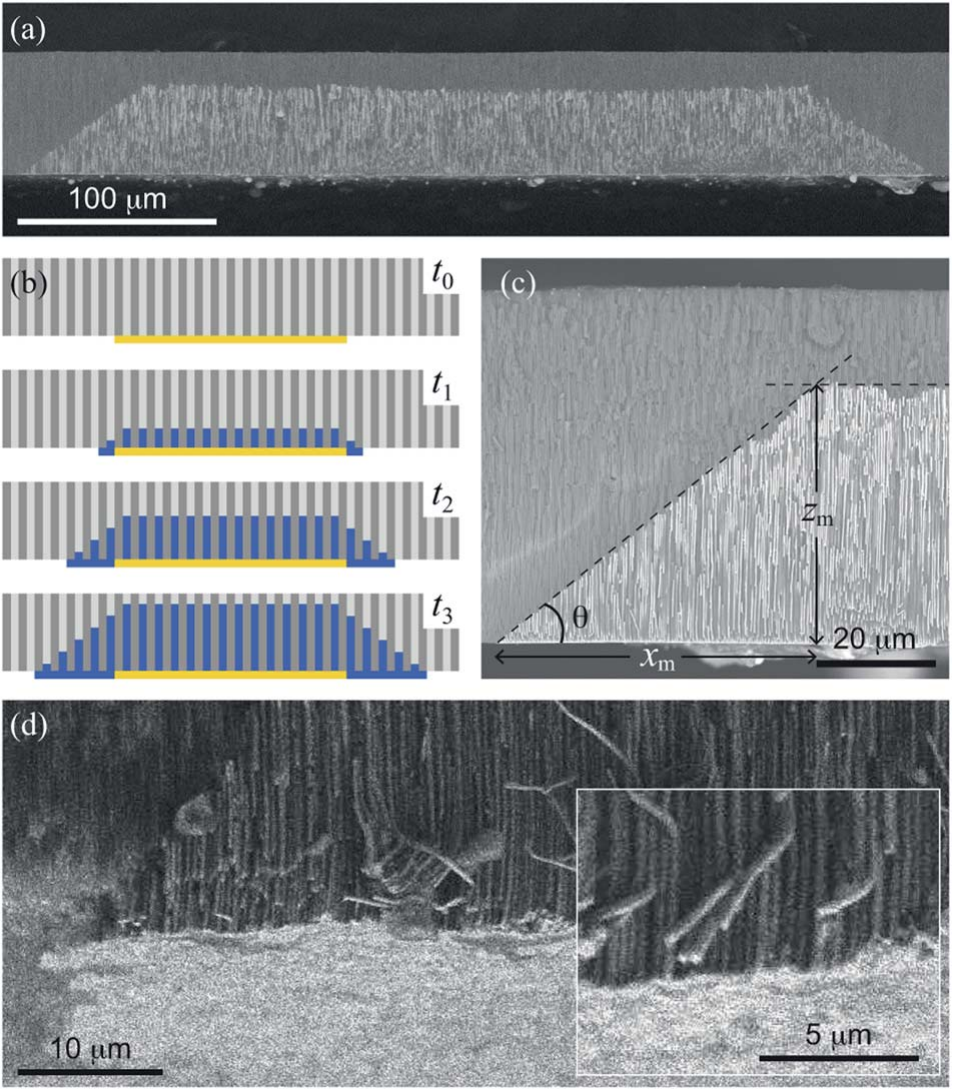
(a) SEM micrograph of the cross section view of an array of Ni NWs with two μHGPs grown above a 330 μm wide EGaIn cathode using the Ni-1.0 M electrolyte at −1.0 V. (b) Schematics of a series of stages that take place in the sequential times *t*_0_ < *t*_1_ < *t*_2_ < *t*_3_ for the μHGP growth mechanism starting from an EGaIn cathode painted beneath the AAO membrane. (c) SEM micrograph of a close view of a Ni μHGP grown using the Ni-1.0 M electrolyte at −1.0 V, displaying its dimensions determined using the two tangent method represented by the intersection of the dashed lines. (d) SEM micrograph of an inclined view displaying both the μHGP (dark contrast) and the generated metallic layer (bright contrast) beneath the NWs. The inset in this figure is a close view displaying sharp details of the bottom metallic layer.

Micro-height gradient profiles are novel structures that consist of a gradual variation of the NWs height along the in-plane or horizontal direction from the EGaIn cathode edges, as shown in Fig. 2(a) for an array of Ni NWs grown using the corresponding Ni-1.0 M solution at −1 V. As expected, NWs with the same height grow above the painted zones with EGaIn since their nucleation begins at the same time. Further insight on the μHGP growth mechanism is provided by the schematics shown in Fig. 2(b). Prior to the beginning of the growth, in the first stage at time *t*_0_ the reduction potential is established between the counter electrode connected to the EGaIn cathode and the working electrode. In the second stage at time *t*_1_, the NWs begin their nucleation inside the AAO membrane pores, which is accompanied by a thin metallic layer that grows from the edges of the EGaIn cathode along the in-plane direction.

This lateral growth is induced by the electrical contact between the edges of the EGaIn cathodes with the electrolyte, so the progressive lateral extension of the EGaIn cathodes creates new nucleation sites for other NWs. In the third and subsequent stages at times *t* ≥ *t*_2_, the uninterrupted growth of the NWs and the creation of new nucleation sites at the cathode result in NWs with different heights because their nucleation take place at different times. Linear or trapezoid-like μHGPs are obtained when both the NWs and the metallic layer in the in-plane direction grow at a constant rate, as seen in Fig. 2(c). The angle *θ* of a μHGP can be determined by using the two tangent method, which locates the point beneath the intersection of the two fitting lines to both, the horizontal and inclined sides of the trapezoid-like structure made of NWs. This point serves to measure the maximum lateral extension (*x*_*m*_) of the μHGP which leads to *θ* = tan^−1^(*z*_*m*_/*x*_*m*_) where *z*_*m*_ is the maximum NWs height. The *y*-coordinate lies along the depth of the μHGP structure.

An interesting feature of the metallic layer grown beneath the μHGP shown as bright contrast in Fig. 2(d) is its very low thickness that is comparable to the NWs diameter. The close magnification view displayed in the inset of this figure provides further evidence of the very low thickness of such a metallic layer (bright contrast) at the bottom of the NWs. The fact that this layer is considerably thin can be explained by the electric field established between the EGaIn layers and the Pt working electrode which is strong enough to avoid the migration of ionic species at the bottom of the EGaIn cathode. This very thin layer is not the main structure, however, it plays a fundamental role in the growth of a μHGP and can easily be removed if desired.

### 3.2 Morphology of μHGPs

**Fig. 3:**
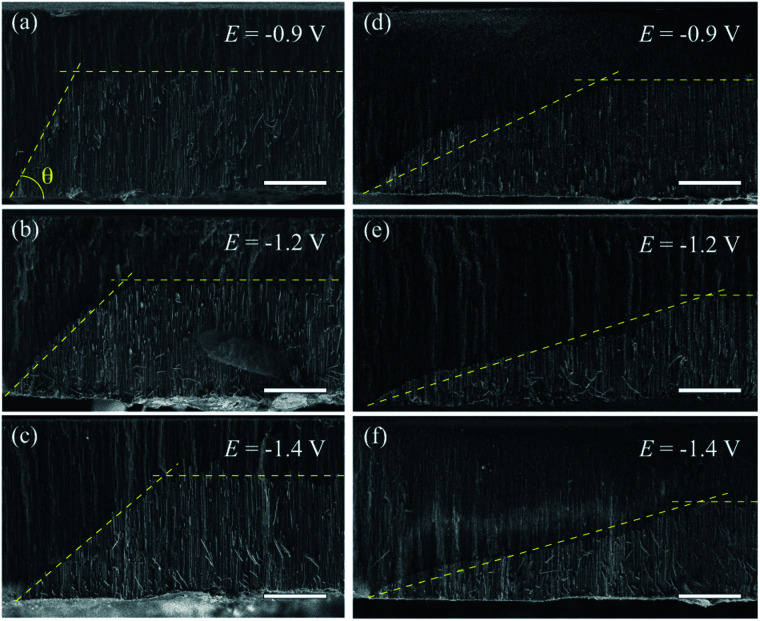
SEM micrographs of the cross section view of (a–c) Ni and (d–f) Co μHGPs grown from 0.5 M electrolytes at the constant reduction potentials of −0.9 V, −1.2 V and −1.4 V. The dashed lines are linear fits used to approximate the μHGPs shape to a trapezoid-like geometry which is used along with the two tangent method to determine their dimensions and the inclination angle *θ*. In all figures scale bars represent 20 μm.

Previous studies suggest a dependence of the NWs growth rate on the reduction potential.^47^ Therefore, different inclination angles *θ* for μHGPs grown at different constant reduction potentials are expected. Indeed, clear morphological changes are evidenced for both Ni and Co μHGPs grown from 0.5 M electrolytes at the constant reduction potentials of −0.9 V, −1.2 V, and −1.4 V, as shown respectively in Fig. 3(a–c) and (d–f). Although deviations from the expected linear profile, like the one shown in Fig. 2(c), are more visible for Co μHGPs, the main effect of decreasing the average angle *θ* as |*E*| increases is clearly maintained for both NW materials. Conversely, the situation is reversed at higher reduction potentials close to −1.4 V, being consistent with a faster appearance of new nucleation sites along the lateral direction than the growth of NW inside the pores.

The different morphologies observed for Ni and Co μHGP at a fixed *E* value suggests that the growth dynamics, through the ionic mobility of each material, plays a fundamental role in their growth. This feature can be ascribed to the different interactions between the metallic cations in solution and the electric potential originated by the surface-bound hydroxyl (–OH) groups at the AAO membrane pore walls which make them hydrophilic.^48^ This effect depends on other factors like the electric double layer or Debye length, the pore diameter, the nature and concentration of cations, and the solution pH.^49^ Particularly, the Debye length is in the range 1–50 nm and can influence the cations transport in aqueous solutions through nanopores with diameters as large as 200 nm.^50^ The electrostatic screening has a greater influence inside pores of lower diameter due to electrokinetic effects.^51^

Therefore, due to these effects the cations transport inside the pores is expected to be different from the cations transport in the free solution at the bottom metallic layer growing laterally beneath the NWs.

**Fig. 4:**
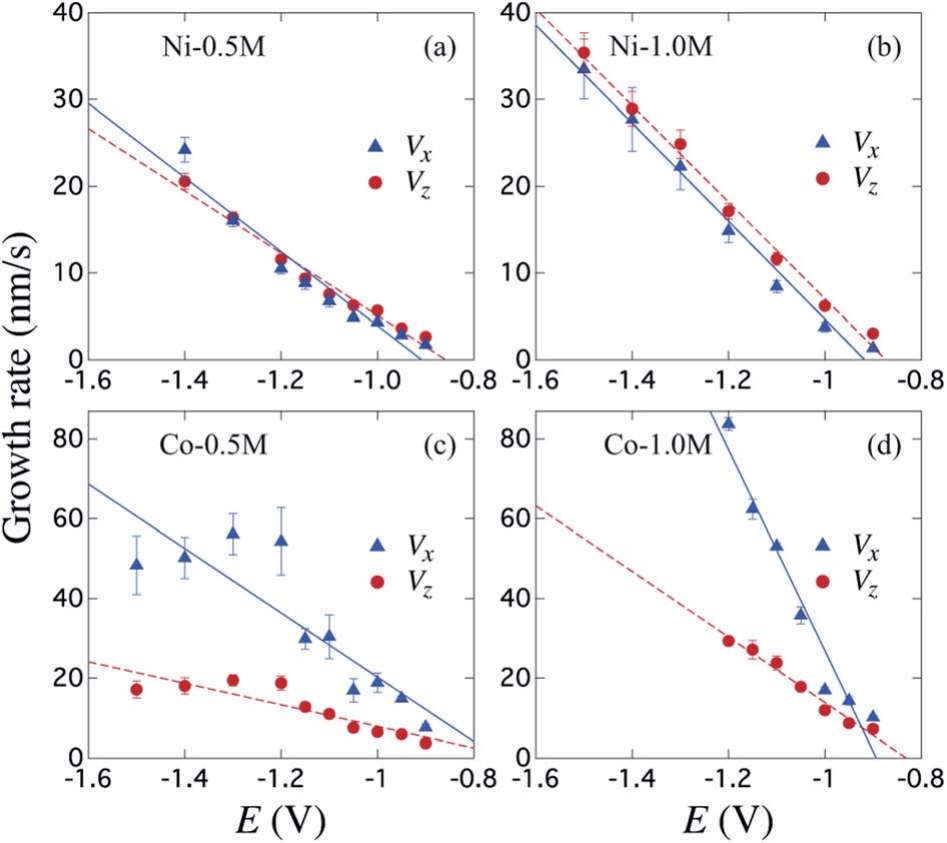
Variation of the metallic layer and NWs average growth rates *V*_*x*_ (triangles) and *V*_*z*_ (circles) for μHGPs grown using the (a) Ni-0.5 M, (b) Ni1.0 M, (c) Co-0.5 M and (d) Co-1.0 M electrolytes as function of the reduction potential *E*. The continuous and dotted lines are linear fits to the data and error bars correspond to one standard deviation obtained from the different μHGPs of a same sample.

Fig. 4 shows the variation of the bottom metallic layer and NWs average growth rates *V*_*x*_ (triangles) and *V*_*z*_ (circles) as a function of the reduction potential *E*. These parameters have been obtained using the two tangent method as in Fig. 2(c), thus averaging the dimensions of the different μHGPs in the same porous membrane and dividing them by the total growth time. Constant growth rates are better suited than the μHGPs dimensions for their morphological characterization and comparison between each other because they have neither the same growth time nor the same maximum height. Error bars to the data correspond to one standard deviation resulting from the dimensions dispersion of the μHGPs in a single AAO membrane with four EGaIn lines. A general behavior is the increase of both *V*_*x*_ and *V*_*z*_ with increasing |*E*|. Specifically, increasing the metallic cation concentration for a specific material and at a fixed *E* value leads to slightly larger growth rates in both directions. As observed from the comparison between Fig. 4(a) and (b) for the case of Ni μHGP, these parameters behave similarly as *E* increases, with *V*_*z*_ being slightly larger than *V*_*x*_ for both electrolytes. At low |*E*| values, both vertical and horizontal growths take place in a slow cation transport regime with the migration of mainly metallic species. However, during electrodeposition both reduction of metallic cations and hydrogen evolution reactions (HER) take place, leading as a result to a reduced cathodic current efficiency for the NWs growth.^52,53^ A further increase of the reduction potential can lead to an increase of the current density for the HER which in turn can have a significant impact in decreasing the NWs cathodic current efficiency.^47^ Therefore, larger reduction potentials can be responsible for more limited growths along the vertical direction. This mechanism can explain the morphological changes that lead to a decrease of the μHGPs angle with increasing |*E*|, as observed in Fig. 3(a–c).

### 3.3 Influence of electrochemical parameters on the μHGPs growth

On the other hand, a significant potential-dependent difference between *V*_*x*_ and *V*_*z*_ for Co μHGPs grown using both 0.5 M and 1.0 M electrolytes is observed in Fig. 4(c) and (d). The fact that *V*_*x*_ > *V*_*z*_ leads to angles lower than 45° as observed in Fig. 3(d–f) for Co μHGPs. Still lower inclination angles are expected for the Co μHGPs grown using the 1.0 M electrolyte since very large *V*_*x*_ values take place at reduction potentials up to −1.2 V. The non-monotonous behavior along with large error bars for *V*_*x*_ at |*E*| > 1.2 V observed in Fig. 4(c) evidence an unstable growth that leads to a non-negligible dispersion in the μHGPs morphology. Although Co μHGPs can be grown in the potential range from −0.9 V to −1.5 V using the Co-0.5 M electrolyte, only stable growths in the range from −0.9 V to −1.2 V can be achieved using the Co-1.0 M electrolyte. Increasing further the potential with the highly concentrated electrolyte does not allow to obtain well defined μHGPs because the corresponding very large growth rates facilitate the HER.

Besides, the growth rates in Fig. 4 fairly display a linear behavior in most of the cases as shown by the continuous and dotted lines fitted to the data, which can be expressed in the slope-intercept form as *V*_*x*_ = *m*_*x*_*E* + *b*_*x*_ and *V*_*z*_ = *m*_*z*_*E* + *b*_*z*_. Since these growth rates are average velocities computed by dividing the μHGPs dimensions into the horizontal and vertical directions by the growth time, they can be expressed as *V*_*x*_ = *x*/*t* and *V*_*z*_ = *z*/*t* for a particular time *t*. Combining these expressions with the slope-intercept linear equations given above leads to the NWs height *z* as a function of the lateral position *x* measured from the endpoint of the μHGP base, that is1
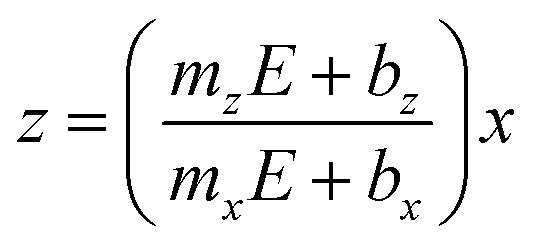


Eqn (1) reproduces the straight line fitted to the inclined side of the trapezoid-like μHGP at a specific reduction potential value. Particularly, since the slope of this line depends on the reduction potential, it directly leads to the potential-dependent inclination angle2
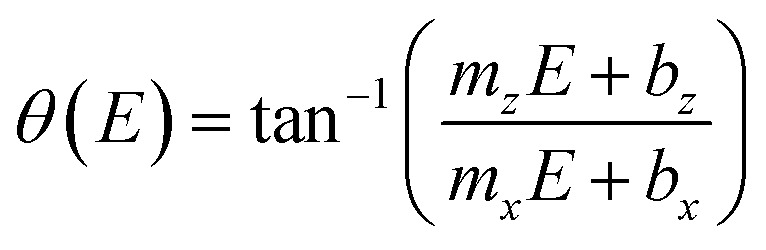


This equation predicts the morphology of a linear μHGP synthesized using a specific electrolytic solution and at particular constant reduction potential. The slope (*m*_*i*_) and *z*-intercept (*b*_*i*_) coefficients for *i* = *x*, *z* can be used as input parameters in eqn (2) to obtain the variation of each series of μHGPs, as they depend on the cations material and the electrolyte concentration. Table 1 summarizes the as-obtained values for *m*_*i*_ and *b*_*i*_ for the different electrolytes, given in units of nm s^−1^ V^−1^ and nm s^−1^, respectively. The inclination angle of each μHGP has been determined as *θ* = tan^−1^(*z*_*m*_/*x*_*m*_) and compared with the variation of *θ*(*E*) *vs. E* to validate eqn (2).

**Table 1:** Slope (*m*_*i*_) and *z*-intercept (*b*_*i*_) values of the linear fits performed to the growth rates *V*_*i*_ with *i* = *x*, *z* for the four series of μHGPs shown in Fig. 4

Electrolyte	*m*_*x*_ (nm s^−1^ V^−1^)	*b*_*x*_ (nm s^−1^)	*m*_*z*_ (nm s^−1^ V^−1^)	*b*_*z*_ (nm s^−1^)
Ni-0.5 M	−42.71	−38.80	−35.93	−30.84
Ni-1.0 M	−59.49	−51.81	−55.65	−48.62
Co-0.5 M	−80.72	−60.49	−26.98	−19.07
Co-1.0 M	−252.14	−225.26	−82.22	−68.33

**Fig. 5:**
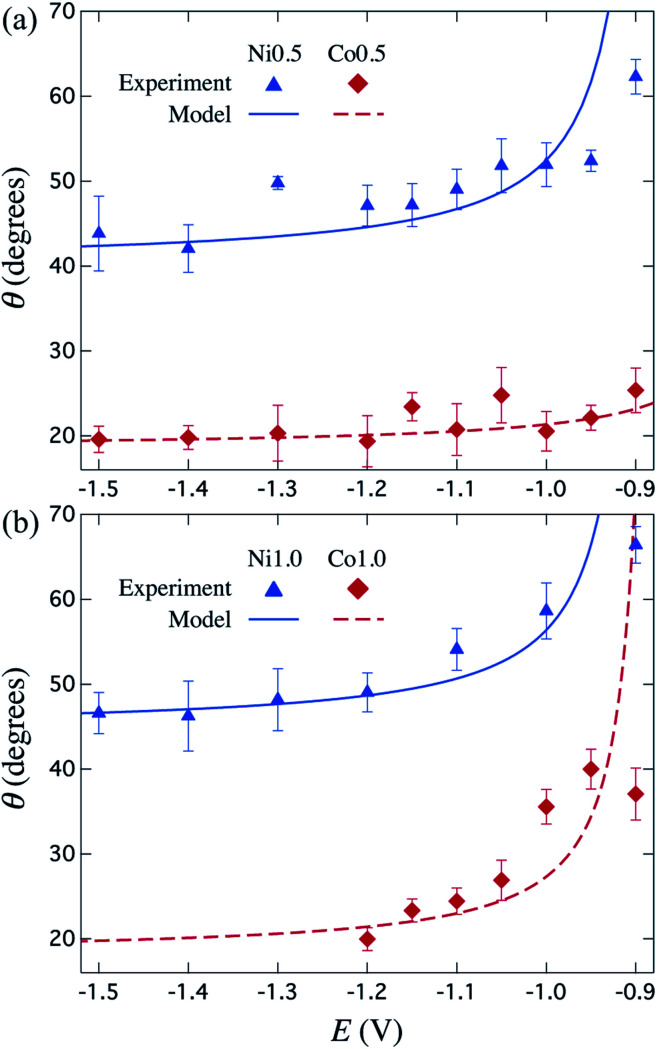
Measured (symbols) and calculated (lines) inclination angle *θ* for Ni (triangles) and Co (lozenges) μHGPs grown at different constant reduction potentials using electrolytes with metal cation concentrations of (a) 0.5 M and (b) 1.0 M. The calculated curves are determined using eqn (2) with the growth rate slope and *z*-intercept coefficients given in Table 1.

Fig. 5 shows a very good agreement between the experimental data and the calculated variation of *θ vs. E*. For each series of μHGPs, *θ* varies by about 20° in the corresponding range of *E* values. However, such angle variations take place in different ranges of values for each series of μHGPs.

Considering all combinations of NWs materials and electrolyte cation concentrations lead to a *θ* variation in a wide range from about 18° to 67°. These angles are much larger and are in a wider range of values than those in the range 1–6° reported in a previous work for NW arrays with height gradient profiles made by a combined dip-coating and electrodeposition method.^30^ As a result, the method proposed in this work has the advantage of obtaining μHGPs whose geometry can be modified more drastically. Another interesting feature of the behavior of *θ* is that larger inclination angles arise at lower reduction potentials for all series of μHGPs for which the electrodeposition process is very slow. At these potentials, eqn (2) predicts higher inclination angles than the measured ones and zero lateral growth for *E* close to −0.9 V. Therefore, although the proposed model provides a pretty good explanation for the behavior of the inclination angle, it is limited to low *E* values because the lateral growth is likely to take place as long as the NWs growth occurs. Furthermore, Ni μHGPs show larger inclination angles than those for Co μHGPs at a fixed cation concentration, indicating higher growth rates along the vertical direction than in the horizontal one using Ni electrolytes. As seen from the comparison between Fig. 5(a) and (b), larger *θ* values up to about 40° are obtained for Co μHGPs grown at low potentials using more concentrated electrolytes. In contrast, *θ* shows a weaker dependence on the cation concentration for the case of Ni μHGPs. The slight variation of both the experimental and predicted *θ* at large |*E*| values corroborate the fact that the growth rates in the vertical and horizontal directions increase proportionally. Therefore, deviations from the linear behavior observed in Fig. 4 are very likely due to the increase of the HER.

### 3.4 Control of the morphology by a variable reduction potential

**Fig. 6:**
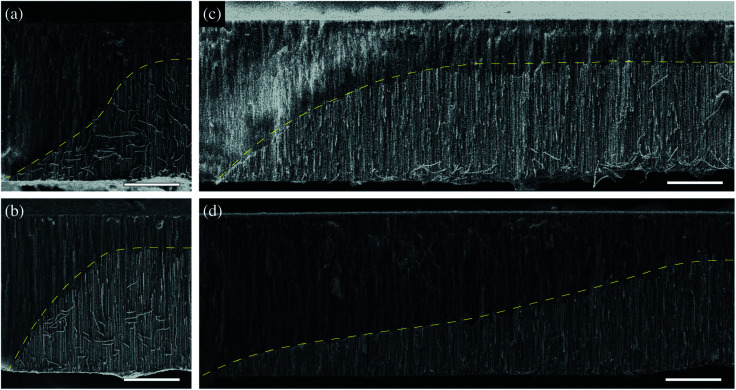
Non-linear (a and b) Ni and (c and d) Co μHGPs obtained by varying the potential using eqn (3) with *p* = 4 for electrolytes with 0.5 M metal cation concentrations and varying the reduction potential in the range (a and c) −0.9 V to −1.4 V and (b and d) −1.4 to −0.9 V. The dashed lines are guides to the eye to highlight the μHGP morphology and scale bars in all figures represent 20 μm.

The dependence of *θ* on *E*, shown in Fig. 5; is the result of the competition between the growth rates in the vertical and horizontal directions. This effect can be exploited for the design of μHGPs with a more complex morphology than the simplest linear one observed for Ni μHGPs grown at constant reduction potentials. This means that to obtain μHGPs with non-linear morphologies or with an enhanced growth along a specific direction, the reduction potential can no longer be constant in time. In order to ensure a non-linear morphology, consider a simple power function for the reduction potential *vs.* time, as3
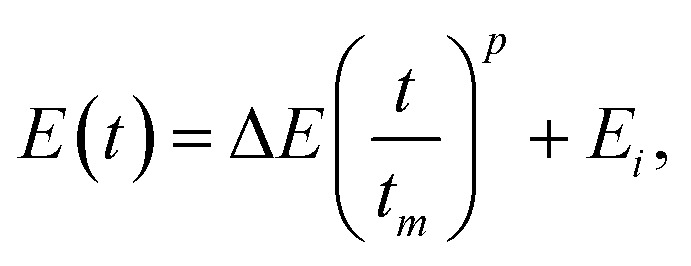
where *p* > 0 is a constant exponent, Δ*E* = *E*_*f*_ − *E*_*i*_ is the difference between the initial (*E*_*i*_) and final (*E*_*f*_) reduction potentials and *t*_*m*_ is the maximum time of the experiment. The main feature of Ni μHGPs grown at constant reduction potentials is the trapezoid-like morphology with a well defined linear inclined side, as observed in Fig. 3(a–c). Therefore, using eqn (3) to vary the reduction potential can surely induce deviations from the linear morphology of these μHGPs. In order to carry out the variation of *E*(*t*) according to eqn (3), a homemade computer program has been used to control the SMU. The non-linear variation of *E*(*t*) given by the exponent *p* is not enough to define a specific non-linear morphology because the extreme potential values can be chosen in two different ways. Choosing |*E*_*i*_| < |*E*_*f*_| or |*E*_*i*_| > |*E*_*f*_| with *E*_*i*_ and *E*_*f*_ equal to either −0.9 V or −1.4 V leads to different morphologies, as shown in Fig. 6 (a) and (b) for Ni μHGPs. These structures have been grown using the same exponent *p* = 4 and the same extreme values for the interval of the varying potential. The only difference between them is the choice of the magnitude of *E*_*i*_ with respect to *E*_*f*_.

For the μHGP of Fig. 6(a)*E*_*i*_ = −0.9 V and *E*_*f*_ = −1.4 V, so the reduction potential is increasing or accelerating with time, which is responsible for the observed concave morphology. As seen in Fig. 3(a–c) and 5(a), larger reduction potentials favor the horizontal growth that leads to lower inclination angles. This mechanism can explain the preferred concave morphology with a low initial angle as a result of the application of larger potentials at the end of the NWs growth provided that the vertical and horizontal growth rates are similar to each other. Conversely, for the μHGP of Fig. 6(b)*E*_*i*_ = −1.4 V and *E*_*f*_ = −0.9 V, leading as a result to a decelerating growth which is responsible for the observed convex morphology, as expected. In this case, the higher starting potential which is characteristic of lower inclination angles does not have a significant influence on the morphology of the μHGP because it is followed by lower potentials over time which favor larger inclination angles. As a consequence, two different growths are obtained by just switching the initial and final reduction potentials in the experiment.

On the other hand, although eqn (3) has been designed to induce deviations from the linear morphology of Ni μHGPs, it can also be used to obtain Co μHGPs with different morphologies. Both growths of Co μHGPs with |*E*_*i*_| < |*E*_*f*_| and |*E*_*i*_| > |*E*_*f*_| for the accelerating and decelerating regimes, respectively, have been carried out by following the same procedure as for Ni μHGPs. Fig. 6(c) and (d) show Co μHGPs grown considering *p* = 4 for the accelerating and decelerating potential regimes. As seen, the morphology of these structures is now convex- and concave-like, respectively, which is contrary to what is obtained for the Ni μHGPs grown under the same conditions. In order to understand this change of morphology, observe from the comparison between Fig. 4(a, b) and (c, d) that a potential-dependent gap between *V*_*x*_ and *V*_*z*_ takes place for Co μHGPs but not for Ni μHGPs. In the accelerating regime (|*E*_*i*_| < |*E*_*f*_|), longer periods take place in a slower growth which is characteristic of larger inclination angles [see Fig. 3(d)]. This situation can explain the convex-like morphology of the structure. At this stage, the gap between *V*_*x*_ and *V*_*z*_ is not too important, so new nucleation sites are created in a controlled way, thus limiting to some extent their accelerated generation. However, as observed; the sudden final acceleration and the further increase of *V*_*x*_ with respect to *V*_*z*_ favors the lateral extension of the Co μHGP width. These features are then responsible for the convex and more extended morphology in contrast to what is obtained for Ni μHGPs fabricated under the same conditions.

Conversely, in the decelerating regime (|*E*_*i*_| > |*E*_*f*_|), longer periods take place in a faster growth, which is characteristic of lower inclination angles [see Fig. 3 (f)]. At this stage, *V*_*x*_ > *V*_*z*_ with a significant difference between them, thus promoting a fast creation of new nucleation sites that significantly extend the width of the μHGP. Since most of the time is devoted to larger reduction potentials, it follows that the total growth time is shortened in comparison to that in the accelerating regime. Then, a preferred concave-like morphology is generated instead of the convex-like one. The lower reduction potentials at the end do not have a significant impact on the μHGP morphology because the structure is mainly completed in the initial times as a result of the corresponding faster growth.

On the other hand, by comparing the morphologies of the μHGPs shown in Fig. 3(d) and 6(d), it seems that a more horizontally extended structure is generated when *E* varies in a decelerating way than when *E* is very high but kept constant during the total growth time. Indeed, the average angle of the μHGP of Fig. 3(d) is about 20°, thus larger than the value of about 13° for the μHGP of Fig. 6(d). The lower angle observed when the potential decelerates is due to the longer time required for the NW growth, in contrast to the shorter time when the growth occurs at the highest constant potential. This feature can be explained by the fact that *θ* is nearly independent on *E* for the Co-0.5 M electrolyte [see Fig. 5(a)], whereas *V*_*z*_ clearly depends on |*E*|. The decreasing of *V*_*z*_ in the decelerating regime leads to a growth time delay with respect to the faster growth done at the constant highest reduction potential.

The careful variation of the reduction potential through simple functional expressions can generate broad μHGP morphologies with lateral dimensions that span in the range of 10 to 10^2^ μm. By following the experimental approach reported in this work allows synthesizing not only very narrow microstructures but also the generation of laterally extended structures comparable to those obtained by more complex fabrication techniques like dip-coating.^30^ Besides, the proposed method in this work has the drawback of generating gradients around isolated regions without electrical contact in contrast to heterogeneous nanostructures manufactured by other methods such as two-photon lithography and electron beam-induced lithography.^54,55^ However, its main advantages are its low cost, ease of production, and flexibility to design patterned electrodes useful for a wide variety of potential applications that require 3D structural control.

Finally, the method developed in this work is a novel approach to modulate geometrical features in three dimensions at the micron-scale since it takes advantage of a mechanism that has not been previously identified. The study highlights the role and effects of the main working parameters like the dependence on the reduction potential and cation concentration of the morphology of the μHGPs. Simple control mechanisms are proposed to vary continuously and in a controlled way the deposition potential in order to obtain predetermined morphologies. The method also highlights the dynamical lateral growth of the electrode and its role in the electrochemical reduction of the metal along the perpendicular direction. In this sense, the use of patterned geometries as electrodes can make it possible to control and design three-dimensional shapes of nanowire arrays. Moreover, the combination of the method of this work with micropatterning tools can be used as a different approach to conventional micro-lithography for the fabrication of arrays of nanowire bundles with predesigned shapes. The method also complements currently known processes for modulating the shape and micrometer-scale geometric features of NW template-assisted electrochemical growth in three dimensions. Overall, this method can be adapted for many of the NW growth variations based on the usual continuous electrode approach, like diameter modulation in anodic aluminum oxide membranes,^56^ multilayered NWs,^57^ and interconnected NWs,^58^ to produce novel 3D modulated NW arrays.

## 4 Conclusions

A novel experimental methodology for the growth of NW-based 3D microstructures with controlled μHGPs morphology has been developed. The growth mechanism of these microstructures is the result of a 3D growth caused by the electrical contact between the electrolyte and the edges of the EGaIn cathode located at the bottom side of the AAO membrane. Specifically, the very thin metallic layer that progressively grows from the EGaIn cathode along the horizontal direction generates new nucleation sites which promote the growth of new NWs at different starting times. It has been found that both the material and concentration of metallic cations have a direct impact on the μHGPs growth. That is, the type of cation material leads to a different lateral span or cross-section width of the μHGPs, such that using Co-based electrolytes lead to more laterally extended μHGPs than using Ni based electrolytes. Besides, increasing the cation concentration for both Co and Ni μHGPs has the influence of slightly reducing their lateral span or increasing their average inclination angle. Furthermore, the reduction potential is the main electrochemical parameter influencing the μHGPs morphology. Indeed, both the NW and the growth rates of the thin metal layer have been found to increase significantly with the magnitude of the reduction potential. As a result, a strong dependence of the μHGPs inclination angle with the potential has been observed. This feature has been used for the design of μHGPs with more complex morphologies than the simplest linear one observed mainly at constant reduction potentials. Accelerating or decelerating the reduction potential as a function of time leads to interesting morphologies as convex- and concave-like, as well as very horizontally extended μHGPs. Overall, this methodology is reliable for synthesizing 3D NW-based microstructures with fine-tuned shapes that are interesting for non-reciprocal microwave absorption and super-capacitive applications, which require a very large surface area and controlled morphology.

## Author contributions

Juan Patiño Cárdenas: methodology, validation, formal analysis. Armando Encinas: supervision, writing – review & editing, funding acquisition, investigation. Rossana Ramírez Villegas: investigation, data curation, validation. Joaquín de la Torre Medina: conceptualization, funding acquisition, investigation, project administration, software, writing – original draft.

## Conflicts of interest

There are no conflicts to declare.

## Supplementary Material
